# Anthocyanin Characterization, Total Phenolic Quantification and Antioxidant Features of Some Chilean Edible Berry Extracts 

**DOI:** 10.3390/molecules190810936

**Published:** 2014-07-28

**Authors:** Anghel Brito, Carlos Areche, Beatriz Sepúlveda, Edward J. Kennelly, Mario J. Simirgiotis

**Affiliations:** 1 Laboratorio de Productos Naturales, Departamento de Química, Facultad de Ciencias Básicas, Universidad de Antofagasta, Av. Coloso S-N, Antofagasta 1240000, Chile; E-Mail: Anghel.Brito@gmail.com; 2 Departamento de Química, Facultad de Ciencias, Universidad de Chile, Casilla 653, Santiago 7800024, Chile; E-Mail: areche@uchile.cl; 3 Departamento de Ciencias Químicas, Universidad Andrés Bello, Campus Viña del Mar, Quillota 980, Viña del Mar 2520000, Chile; E-Mail: bsepulveda@uc.cl; 4 Department of Biological Sciences, Lehman College and The Graduate Center, The City University of New York, 250 Bedford Park Boulevard West, Bronx, NY 10468, USA; E-Mail: kennelly@lehman.cuny.edu

**Keywords:** endemic berries, poliphenolics, quantitation, antioxidants, HPLC-MS, murtilla, calafate, chequen, arrayan, meli, luma, blueberry

## Abstract

The anthocyanin composition and HPLC fingerprints of six small berries endemic of the VIII region of Chile were investigated using high resolution mass analysis for the first time (HR-ToF-ESI-MS). The antioxidant features of the six endemic species were compared, including a variety of blueberries which is one of the most *commercially significant* berry crops in Chile. The anthocyanin fingerprints obtained for the fruits were compared and correlated with the antioxidant features measured by the bleaching of the DPPH radical, the ferric reducing antioxidant power (FRAP), the superoxide anion scavenging activity assay (SA), and total content of phenolics, flavonoids and anthocyanins measured by spectroscopic methods. Thirty one anthocyanins were identified, and the major ones were quantified by HPLC-DAD, mostly branched 3-*O*-glycosides of delphinidin, cyanidin, petunidin, peonidin and malvidin. Three phenolic acids (feruloylquinic acid, chlorogenic acid, and neochlorogenic acid) and five flavonols (hyperoside, isoquercitrin, quercetin, rutin, myricetin and isorhamnetin) were also identified. Calafate fruits showed the highest antioxidant activity (2.33 ± 0.21 μg/mL in the DPPH assay), followed by blueberry (3.32 ± 0.18 μg/mL), and arrayán (5.88 ± 0.21), respectively.

## 1. Introduction

**Figure 1:**
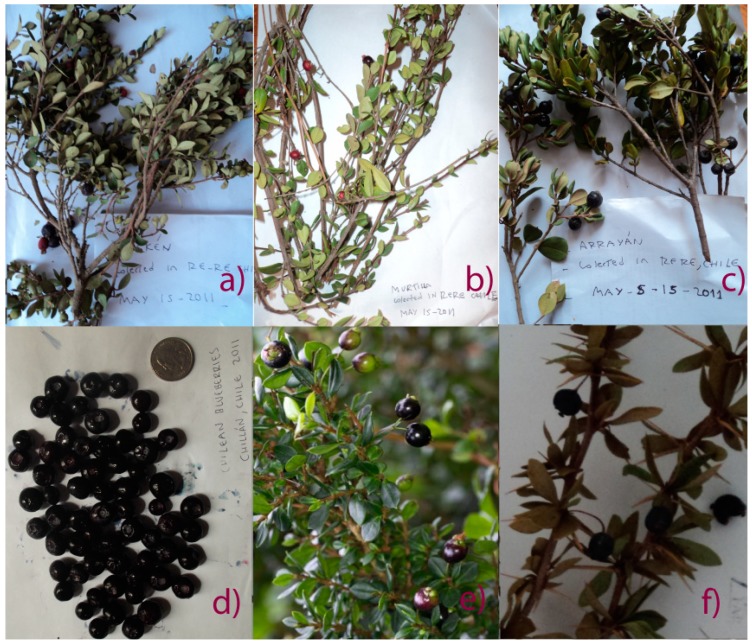
Pictures of (**a**) chequén, (*Luma chequén*) (**b**) murta, (*Ugni molinae*) (**c**) arrayán, (*Luma apiculata*), (**d**) blueberries, (*Vaccinium corymbosum*) (**e**) meli, (Amomyrtus meli and (**f**) calafate (*Berberis microphylla*) growing in the VIII region of Chile.

Fruits and vegetables are considered highly protective for human health, particularly against ageing and various oxidative-stress related diseases, due to their content of healthy phytochemicals [1]. Several epidemiological studies have highlighted the association between the consumption of foods with high contents of phytochemicals, mainly flavonols, phenolic acids and anthocyanins, and the prevention of degenerative diseases such as cardiovascular diseases, ageing, cancer and other degenerative disorders [2,3]. Anthocyanins are a group of red, purple, violet and blue water soluble polyphenolic pigments widely distributed in berry fruits which can act as antioxidants or free radical scavengers, thus preventing oxidative stress [4]. The term berry fruit generally refers to some small fruit that lacks big seeds and can be eaten whole. Berry fruits are often the richest source of antioxidant phytochemicals among fruits and vegetables [5], thus the chemical study of native berry fruits is of great economic significance since it can support the consumption and commercial activities of gatherers, growers, micro-companies and industries associated with the use of native plants. Chilean fruits such as arrayán, chequen, calafate, meli, maqui and murta (Figure 1) are small pigmented native berries which were collected since pre-Colombian times by South American Amerindians as a food source. At present, there is still some regional consumption of the small berries from trees and shrubs belonging to the Myrtaceae (Chilean myrtle, murta, arrayán, chequén, luma and meli), Berberidaceae (michay and calafate) as well as Eleaocarpaceae (maqui) occuring in southern Chile and Argentina. In Chile, “murta” or “murtilla” (*Myrtus ugni* Molina or *Ugni molinae* Turczaninov), a wild perennial shrub also commonly known as Chilean guava, is the best-known of the native Myrtaceae plants, where the people have long appreciated its red edible berries for its unique aroma. Infusions of the leaves of this species are anti-inflammatory and analgesic [6] and the fruits contain several volatile compounds responsible for the aroma [7]. 

Arrayán (*Luma apiculata* (DC.) Burret is an evergreen Myrtaceae tree occurring in southern Chile and Argentina of about 10 m in height with orange-red trunk and edible purple black berries, 1–1.5 cm in diameter, that ripen in early autumn and are half the size, with more intense color, but similar aspect and consistence as the worldwide commercialized blueberries (*Vaccinium corymbosum*). Murillo [8] describes the medicinal properties of *Eugenia apiculata* D.C. (a synonym for *L. apiculata*, also known as *Myrceugenella apiculata* (DC.) Kausel [9]). The traditional use indications include aromatic, slightly astringent, balsamic and anti-inflammatory uses. The fruits were used to prepare liquor. This information is in agreement with the aromatic flavor that is attractive for local producers of alcoholic beverages. The fruits of *Luma chequén* (Molina) A. Gray, syn: *Myrceugenella chequen* (Mol.) Kaus are edible small berries with similar size than those of arrayán and murta. de Mösbach [10] refers to uses of *L. chequen* in infusions and syrups as an astringent. The traditional use indications in traditional medicine can be related to the tannin content of the plant which is also recommended as a wound wash and to treat dysentery. Both *L. apiculata* and *L. chequen* fruits were used to prepare “chicha”, a South American native fermented beverage [9]. Calafate or Magellan barberry (*Berberis microphylla* G. Forst, sin. *Berberis buxifolia*, and *Berberis heterophylla*) is another Patagonian shrub with edible dark small berries that can grow in a great variety of areas [11]. The production of calafate is concentrated in small gardens in the Regions of Aysén and Magallanes for local production of jams and juices [11]. This fruit contains several anthocyanins [12] and high content of cinnamic acids [13]. Maqui (*Aristotelia chilensis*) fruit is now one of the most famous dark colored Chilean berries because of its high content of anthocyanins [14]. Calafate, maqui and murta are antioxidant berries considered superfruits due to their high content of phenolic compounds, including several anthocyanins [6,12,15]. Several edible Myrtaceae fruits known worldwide present free radical scavenging constituents including anthocyanins [16], while Chilean Myrtaceae with high anthocyanin contents have been assessed for antioxidant activity and showed good antioxidant features [17,18,19]. M*ass*°spectrometry has undergone tremendous *technological improvements* in the last years, especially with the development of ionization methods such as electrospray (ESI), atmospheric pressure chemical ionization (APCI) and high resolution mass detectors such as time of flight (TOF). Indeed, several antioxidant phenolics in edible plants [20]; fruits [21,22,23]; nuts [24] and food byproducts [25] were analyzed using HPLC hyphenated with accurate high resolution time of flight analyzers (HPLC-PDA-ToF-MS). However, the chemical analysis regarding anthocyanins or metabolomics present in wild Chilean berries including arrayán, chequén, murta, and calafate was performed using low resolution methods (ESI-ion trap-MS) [12,15,19], while the phenolic constituents of *A. meli* have not beenreported to the best of our knowledge. 

The aim of the present work was the analysis by high resolution mass spectrometry (HR-MS) of some important native berries from Chile, and the comparison of the antioxidant properties and total phenolics. In the present work the anthocyanin fingerprints and polyphenolic content of six small Chilean berries (arrayán, chequén, murta, calafate, meli and Chilean blueberry var. Brigitta, Figure 1) from the VIII region of Chile were compared and correlated with the antioxidant capacities measured by the DPPH radical bleaching, ferric reducing antioxidant power (FRAP), and the superoxide anion scavenging activity (SA) assays. The anthocyanins in berries were identified for the first time with the help of PDA analysis and high resolution time of flight mass spectrometry (HPLC-ESI-ToF-MS) plus comparison with authentic standards.

## 2. Results and Discussion

### 2.1. Accurate MS-PDA Identification of Anthocyanins in Six Small Berry Fruits from Southern Chile

**Figure 2:**
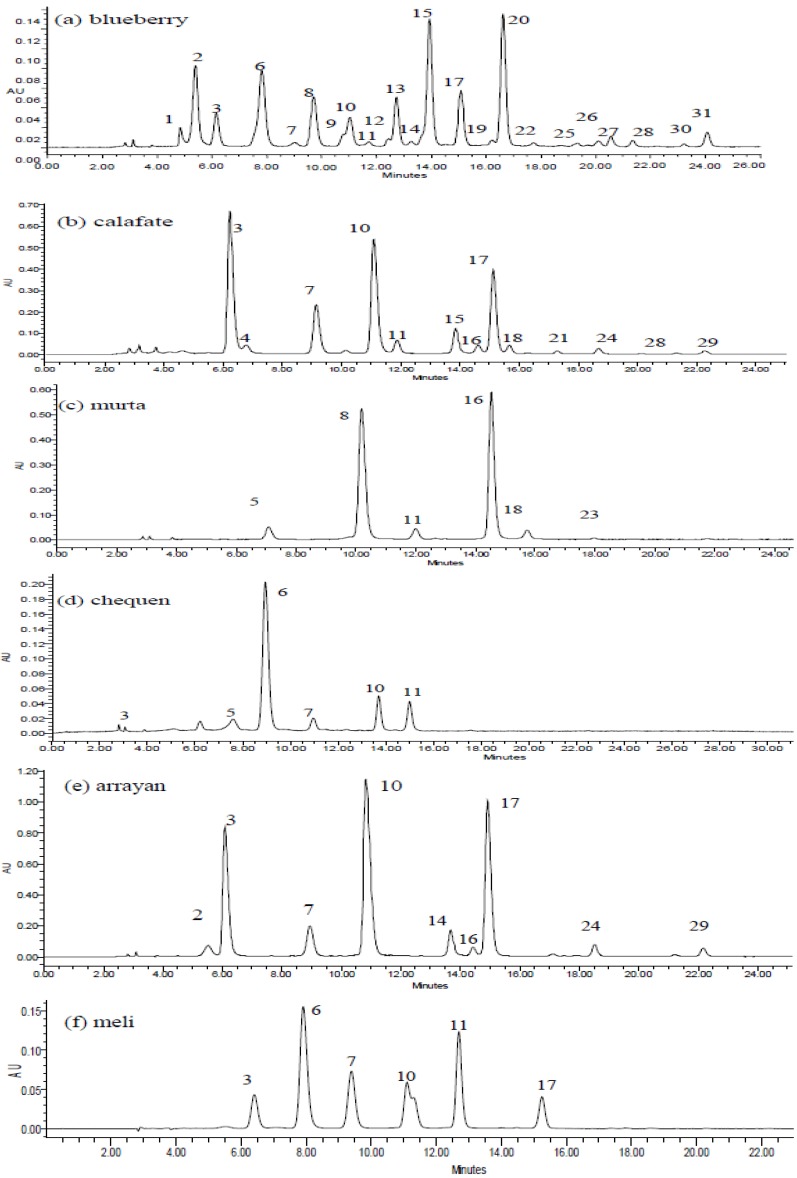
HPLC-PDA chromatograms of six berries from the VIII region of Chile. (**a**) *Vaccinium corymbosum*, (**b**) *Berberis microphylla*, (**c**) *Ugni molinae*, (**d**) *Luma chequén*, (**e**) *Luma apiculata*, and (**f**) Amomyrtus meli monitored at 520 nm. Peaks numbers refer to those indicated in Table 1.

**Figure 3:**
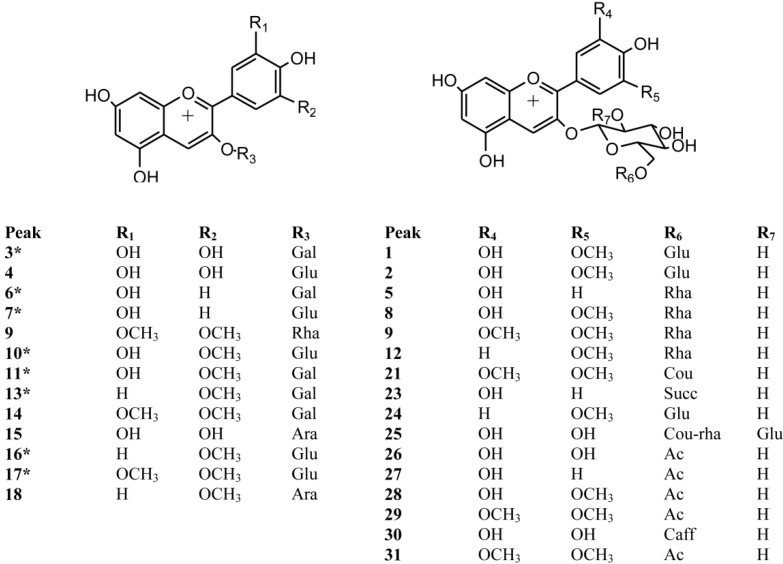
Structures of the anthocyanins identified in six berries from the VIII region of Chile.

Anthocyanins in berry fruits were accurately detected and identified using HPLC with UV-visible detection (PDA, Figure 2, Table 1) and high resolution time of flight mass spectrometry (HR-ToF-MS, Table 1). The 31 anthocyanins identified in the six berries (Figure 3) were mainly 3-*O*-glycoside conjugates and their derivatives.

* Identified using standard compounds. Gal: Galactose; Glu: Glucose; Ara: Arabinose; Rha: Rhamnose; Cou: Coumaric; Succ: Succinic acid; Ac: Acetyl group; Caff: Caffeic acid.

Twenty three compounds were detected in blueberry (peaks **1**–**3**, **6**–**15**, **17**, **19**, **20**, **22**, **25**–**28**, **30** and **31**, Table 1) fourteen in calafate (peaks **3**, **4**, **7**, **8**, **10**, **11**, **15**, **16**–**18**, **21**, **24**, **28** and **29**), nine in arrayán (peaks **2**, **3**, **7**, **10**, **14**, **16**, **17**, **24** and **29**), and six in meli (peaks **3**, **6**, **7**, **10**, **11** and **17**), chequén (peaks **3**, **5**, **6**, **7**, **10** and **11**) and murta (peaks **5**, **8**, **11**, **16**, **18** and **23**). Figure S2 and S3 (Supplementary Material) show as examples full scan ToF-MS spectra of peaks **3**, **8**, **9**, **10**, **16**, **17**, **21**, **22** and **28**). Peaks **3**, **6**, **7**, **10**, **11**, **13**, **16** and **17** were identified by spiking experiments with authentic standards as delphinidin 3-*O*-galactoside (HR-MS ion at *m/z* 465.1043, λ_max_: 276–523), cyanidin-3-*O*-galactoside (HR-MS ion at *m/z* 449.1052, λ_max_: 280–511), cyanidin-3-*O*-glucoside (HR-MS ion at *m/z* 449.1099, λ_max_: 280–517), petunidin-3-*O*-glucoside (HR-MS ion at *m/z* 479.1233, λ_max_: 276–526), petunidin-3-*O*-galactoside (HR-MS ion at *m/z* 479.1233, λ_max_: 276–523), peonidin-3-*O*-galactoside (HR-MS ion at *m/z* 463.1234, λ_max_: 279–520), peonidin-3-*O*-glucoside (HR-MS ion at *m/z* 463.1258, λ_max_: 279–523), and malvidin-3-*O*-glucoside (HR-MS ion at *m/z* 493.1252, λ_max_: 276–527), (Table 1), respectively.

**Table 1:** Identification of phenolic compounds in chilean berries by LC-PDA-HR-ToF-ESI-MS data.

Peak Number	Retention Time (min)	Uv max	HR-M + ion (ppm)	Other ions (Aglycon moiety)	Formula	Identification	Fruit
**1**	4.8	276*–*523	641.1687 (−4.8)	317.0618 (Petunidin)	C_28_H_33_O_17_	Petunidin-3-*O*-di-hexoside	blue
**2**	5.9	280–517	611.1614 (0.3)	449.1709 (Cyanidin-3-*O*-hexoside)	C_27_H_31_O_16_	Cyanidin- 3-*O*-di-hexoside	blue, arr
**3**	6.3	276–523	465.1040 (1.8)	303.0500 (Delphinidin)	C_21_H_21_O_12_	Delphinidin 3-*O*-galactoside *	blue, cal, che, arr, lu
**4**	6.8	276–525	465.1038 (1.1)	303.0495(Delphinidin)	C_21_H_21_O_12_	Delphinidin 3-*O*-glucoside *	cal
**5**	7.1	280–517	595.1478 (−31.0)	449.1089 (Cyanidin-3-*O*-glucoside)	C_27_H_31_O_15_	Cyanidin 3-*O*-rutinose	mu
**6**	7.8	280–511	449.1052 (−7.1)	287.0675 (Cyanidin)	C_21_H_21_O_11_	Cyanidin-3-*O*-galactoside *	blue, che, lu
**7**	9.1	280–517	449.1099 (3.3)	287. 0507 (Cyanidin)	C_21_H_21_O_11_	Cyanidin-3-*O*-glucoside *	blue, che, lu
**8**	9.8	276–526	625.1789 (3.2)	479.1198 (Petunidin-3-*O*-glucoside)	C_28_H_33_O_16_	Petunidin-3-*O*-rutinoside	blue, cal, mu
**9**	10.7	276–527	639.1911 (−2.2)	493.1136 (Malvidin-3-*O*-glucoside)	C_29_H_35_O_16_	Malvidin-3-*O*-rutinose	blue
**10**	11.2	276–526	479.1233 (9.0)	317.0672 (Petunidin)	C_22_H_23_O_12_	Petunidin-3-*O*-glucoside *	blue, che, arr, lu
**11**	11.9	276–523	479.1224 (7.1)	317.0646 (Petunidin)	C_22_H_23_O_12_	Petunidin-3-*O*-galactoside *	blue, cal, mu, che, lu
**12**	12.5	276–525	609.1825 (0.8)	301.0829 (Peonidin)	C_28_H_33_O_15_	Peonidin 3-*O*-rutinose	blue
**13**	12.7	279–520	463.1234 (−1.3)	301.0689 (Peonidin)	C_22_H_23_O_11_	Peonidin-3-*O*-galactoside *	blue
**14**	13.4	276–527	493.1361 (3.0)	331.0832 (Malvidin)	C_23_H_25_O_12_	Malvidin-3-*O*-galactoside *	blue, arr
**15**	14.0	276–523	435.0936 (2.1)	303.0472 (Delphinidin)	C_20_H_19_O_11_	Delphinidin-3-*O*-arabinoside	blue, cal
**16**	14.7	276–527	463.1258 (3.9)	301.1257 (Peonidin)	C_22_H_23_O_11_	Peonidin-3-*O*-glucoside	cal, mu, arr
**17**	15.3	276–527	493.1252 (−19.0)	331.0789 (Malvidin)	C_23_H_25_O_12_	Malvidin-3-*O*-glucoside *	blue, cal, arr, lu
**18**	15.6	279*–*527	433.1131 (−0.92)	301.0709 (Peonidin)	C_21_H_21_O_10_	Peonidin-3-*O*-arabinoside	cal, mu
**19**	16.2	276*–*526	449.1066 (−4.0)	317.1969 (Petunidin)	C_21_H_21_O_11_	Petunidin-3-*O*-arabinoside	blue
**20**	16.7	280*–*517	419.0978 (−1.9)	287. 0696 (Cyanidin)	C_20_H_19_O_10_	Cyanidin-3-*O*-arabinoside *	blue
**21**	17.3	276*–*311*–*527	639.1933 (34.2)	493.1382 (Malvidin-3-*O*-glucoside)	C_32_H_31_O_14_	Malvidin 3-*O*-(6ꞌꞌ coumaroyl) glucoside	cal
**22**	17.8	276*–*527	463.1284 (9.5)	330.1706 (Malvidin)	C_22_H_23_O_11_	Malvidin-3-*O*-arabinose *	blue
**23**	18.0	280*–*517	549.1639 (7.1)	449.1082 (Cyanidin-3-*O*-glucose)	C_25_H_25_O_14_	Cyanidin-3-*O*-(6ꞌꞌ succinoyl)-glucose	mu
**24**	18.6	279*–*523	625.1820 (8.2)	463.0905 (Peonidin-3-*O*- hexoside)	C_28_H_33_O_16_	Peonidin 3-*O*-di hexoside	cal
**25**	19.4	276*–*311*–*523	919.4460 (2.1)	303.0504 (Delphinidin)	C_42_H_47_O_23_	Delphinidin-3-*O*-rutinose (6ꞌꞌ-p-coumaroyl)-2ꞌꞌ-*O*-glucose	blue
**26**	20.0	276*–*523	507.1135 (−0.4)	303.0495 (Delphinidin)	C_23_H_23_O_13_	Delphinidin 3-*O*-(6ꞌꞌ acetyl) glucoside	blue
**27**	20.6	280*–*517	491.1206 (3.6)	287.1232 (Cyanidin)	C_23_H_23_O_12_	Cyanidin 3-*O*-(6ꞌꞌ acetyl) glucoside	blue
**28**	21.4	276*–*526	521.1293 (−0.4)	317.0676 (Petunidin)	C_24_H_25_O_13_	Petunidin 3-*O*-(6ꞌꞌ acetyl) glucoside	blue, cal
**29**	22.3	276*–*527	535.1451 (−0.2)	331.0789 (Malvidin)	C_25_H_27_O_13_	Malvidin 3-*O*-(6ꞌꞌ acetyl) galactoside	cal, arr
**30**	23.2	276*–*321*–*523	627.1393 (−6.8)	287.0743 (Cyanidin)	C_30_H_27_O_15_	Delphinidin-3-*O*-(6ꞌꞌcaffeoyl)-glucose	blue
**31**	24.0	276*–*527	535.1463 (1.5)	331.0673 (Malvidin)	C_25_H_27_O_13_	Malvidin 3-*O*-(6ꞌꞌ acetyl) glucoside	blue

Abbreviations: blue: Blueberry, cal: Calafate, mu: Murta, che: chequén, arr: Arrayán, me: Meli. * Identified by spiking experiments with authentic compounds.

Peaks **4** and **14** were identified as the monoglucosides delphinidin 3-*O*-glucoside and malvidin-3-*O*-galactoside (HR-MS ions at *m/z* 493.1361 and 465.1038, respectively [19,26,27]. Peaks **1**, **2** and **24** showing HR-MS molecular ions at *m/z* 611.1614, 641.1687 and 625.1820 coincident with the formulas C_27_H_31_O_16_ (0.3), C_28_H_33_O_17_ (−4.8) and C_28_H_33_O_16 _ (8.2) were identified as petunidin (λ_max_: 276–523), cyanidin (λ_max_: 280–517), and peonidin (λ_max_: 279–523), dihexosides [12,28]. In a similar manner, peaks **5** (HR-MS at *m/z* 595.1478, C_27_H_31_O_15_, −31.0), **8** (HR-MS at *m/z* 625.1789, C_28_H_33_O_16_, 3.2), **9** (HR-MS at *m/z* 639.1911, C_29_H_35_O_16_, −2.2) and **12** (HR-MS at *m/z* 609.1825, C_28_H_33_O_15_, 0.8) were assigned as cyanidin, petunidin, malvidin and peonidin rutinosides [12,26,29,30]. Peaks **15**, **18**–**20** and **22** (Figure 2) with HR-MS molecular ions at *m/z* 435.0936 (C_20_H_19_O_11_, 2.1), 433.1131 (C_21_H_21_O_10_, −0.92), 449.1066 (C_21_H_21_O_11_, −4.0), 419.0978 (C_20_H_19_O_10_, −1.9) and 463.1284 (C_22_H_23_O_11_) were identified as delphinidin (λ_max_: 276–523), peonidin (λ_max_: 276–527), petunidin (λ_max_: 276–523), cyanidin (λ_max_: 280–517) and malvidin (λ_max_: 276–527) arabinosides, respectively [26,31], While peaks **21** (HR-MS at *m/z* 639.1933, C_32_H_31_O_14_) and **23** (HR-MS at *m/z* 549.1639, C_25_H_25_O_14_) were identified as malvidin 3-*O*-(6ꞌꞌ coumaroyl) glucoside and cyanidin-3-*O*-(6ꞌꞌ succinoyl)-glucose [28,30]. Peak **25** with a molecular ion at *m/z* 919.4460 (C_42_H_47_O_23_) present in blueberries was identified as the complex anthocyanin: delphinidin-3-*O*-rutinose (4ꞌꞌꞌ-*O*-p-coumaroyl)-2ꞌꞌ-*O*-glucose [27,32]. Peaks **26**–**28** and **31** with HR-MS peaks at *m/z* 507.1135 (C_23_H_23_O_13_), 491.1206 (C_23_H_23_O_12_), 521.1293 (C_24_H_25_O_13_), and 535.1463 (C_24_H_25_O_13_), were identified as delphinidin, cyanidin, petunidin, and malvidin 3-*O*-(6ꞌꞌ acetyl) glucosides as reported [27,31], while peak **30** (HR molecular ion at *m/z* 627.1393 coincident with a formula of C_30_H_27_O_15_ (−6.8) was identified as delphinidin-3-*O*-(6ꞌꞌ caffeoyl)-glucose [29]. An isomer of peak **31** (peak **29**, HR-MS ion at *m/z* 535.1451 (C_25_H_27_O_13_, −0.2), was identified as malvidin 3-*O*-(6ꞌꞌ acetyl) galactoside [27,31]. 

### 2.2. Identification of Phenolic Acids and Flavonols

**Figure 4:**
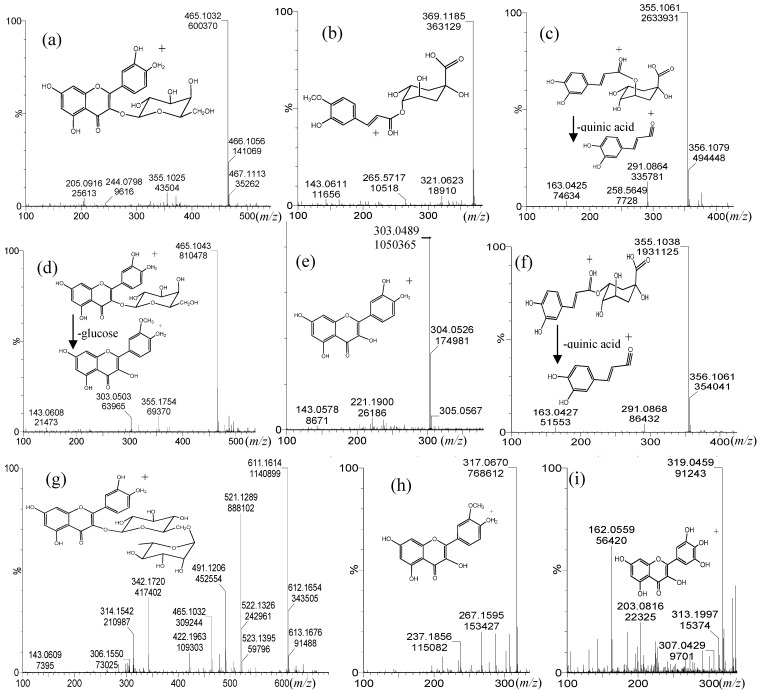
Full scan ToF MS spectra and structures of minor phenolic compounds detected in six berries from the VIII region of Chile. (**a**) Hyperoside, (**b**) feruloyl-quinic acid, (**c**) chlorogenic acid (**d**) isoquercitrin (**e**) quercetin, (**f**) neochlorogenic acid (**g**) rutin (**h**) isorhamnetin and (**i**) myricetin.

Other minor phenolic compounds [12,15,33] were present in all six blueberries analyzed which were accurately identified (Figure 4). The phenolic acids: feruloyl-quinic acid (HR-ToF-MS: 369.1105, MF: C_17_H_21_O_9_, −0.3), chlorogenic acid (HR-ToF-MS: 355.1061, MF: C_16_H_19_O_9_, 9.0) and neochlorogenic acid (HR-ToF-MS: 355.1038, molecular formula: C_16_H_19_O_9_, 2.5), the flavonols quercetin (HR-ToF-MS: 303.0489, MF: C_15_H_11_O_7_, error −5.3), myricetin (HR-ToF-MS: 319.0459, molecular formula: C_15_H_11_O_8_, −1.6) rutin (HR-ToF-MS: 611.1614, MF: C_27_H_31_O_16_, 0.3) hyperoside (HR-ToF-MS: 465.1043, MF: C_21_H_21_O_12_, 2.2) isoquercitrin (HR-ToF-MS: 465.1032, MF: C_21_H_21_O_12_, −0.2) and isorhamnetin (HR-ToF-MS: 317.0670, MF: C_16_H_13_O_7_, 2.8; this last flavonoid was only present in chequén fruits).

### 2.3. Total Phenolics, Flavonoids and Anthocyanin Contents

The total phenolic content (TPC) varied from 5.11 ± 0.18 for chequén to 65.53 ± 1.35 µM Trolox equivalents/g DW for calafate fruits, and showed linear correlation with the antioxidant assays (R^2^ = 0.8755 and R^2^ = 0.9143 for TPC/DPPH and TPC/FRAP assays, respectively, Table 2) the TPC of our sample of calafate showed values two times higher than a Chilean sample from Mañihuales [11] but was close to that reported for a Chilean sample from Faro San Isidro [12]. The total anthocyanin content (TAC) ranged from 1.54 ± 0.05 for chequén to 51.62 ± 1.78 mg cyanidin-3-glucoside/g DW for calafate and showed strong linear correlation with the antioxidant assays (R^2^ = 0.7044 and R^2^ = 0.9914 for TAC/DPPH and TAC/FRAP assays, respectively, Table 2). The total flavonoid content (TFC) showed similar trend, varying from 2.57 ± 0.11 for *L. chequén* to 45.72 ± 2.68 mg quercetin/g DW for *Berberis microphylla.* The TFC showed linear correlation with the antioxidant assays (R^2^ = 0.678 for TFC/DPPH and R^2^ = 0.9856 for TFC/FRAP assays, respectively. The total anthocyanin content for our sample of calafate was close to the values reported for Chilean samples collected in La Junta and Darwin (16.76 mmol/g fresh weigh) and Faro San Isidro (15.44 mmol/g fresh weigh) taking into account conversion factors and 85% water loss (approximately 50.11 and 46.21 mg/g dry weight, respectively) [12]. The levels of anthocyanins in the fruits can explain the different intensity in the color especially for murta, which is red-rose, in comparison with calafate which is purple and blueberry and arrayán which are black (Figure 1).

**Table 2:** Scavenging of the 1,1-diphenyl-2-picrylhydrazyl Radical (DPPH), Ferric Reducing Antioxidant Power (FRAP), Superoxide Anion scavenging activity (SA), Total Phenolic Content (TPC), Total Flavonoid Content (TFC), Total Anthocyanin Content (TAC), and Extraction Yields of Six Edible Berry Fruits From the VIII Region of Chile.

Species	DPPH ^α^	FRAP ^β^	SA ^ο^	TPC ^δ^	TFC ^ψ^	TAC ^χ^	Extraction Yields (%) ^µ^
*Vaccinium corymbosum*	3.32 ± 0.18 *a*	96.15 ± 5.39 *df*	72.61 ± 1.91 *r*	45.86 ± 3.46	18.50 ± 3.75 *p*	21.41 ± 1.65	6.72
*Berberis microphylla*	2.33 ± 0.21 *ab*	124.46 ± 6.54	81.31 ± 2.95 *s*	65.53 ± 1.35	45.72 ± 2.68	51.62 ± 1.78	4.99
*Luma chequén*	12.92 ± 0.30	76.22 ± 3.45 *e*	43.79 ± 2.91 *t*	5.11 ± 0.18 *k*	2.57 ± 0.11 *m*	1.54 ± 0.05	7.39
*Luma apiculata*	5.88 ± 0.21	93.4± 4.68 *dg*	64.22 ± 3.46	27.61± 1.61	12.80± 2.43 *np*	15.24 ± 1.49 *l*	6.34
*Ugni molinae*	10.94 ± 0.32 *c*	81.10 ± 4.58 *ehj*	52.22 ± 1.81 *t*	9.24 ± 0.28 *k*	5.54 ± 0.91 *mo*	6.85 ± 0.10	5.21
Amomyrtus meli	7.46 ± 0.10 *b*	88.29 ± 6.34 *fghi*	56.44 ± 2.32	17.52 ± 0.66	11.76 ± 2.04 *no*	13.33 ±2.69 *l*	4.89
Gallic acid ^ϕ^	1.36 ± 0.22 (7.99 ± 1.29 μM)	148.1 ± 8.35	94.39 ± 1.98	-	-	-	-
Cyanidin 3-*O*-glucoside ^ϕ^	8.47 ± 1.23 *c* (17.47 ± 2.53 µM)	95.48 ± 6.72 *ij*	76.85 ± 1.71 *rs*	-	-	-	-

**^α^** Antiradical DPPH activities are expressed as IC_50_ in µg/mL for extracts and compounds. **^β^** Expressed as µM trolox equivalents/g dry weight. **^ο^** Expressed in percentage scavenging of superoxide anion at 100 µg/mL. **^δ^** Total phenolic content (TPC) expressed as mg gallic acid/g dry weight. **^ψ^** Total flavonoid content (TFC) expressed as mg quercetin/g dry weight. **^χ^** Total Anthocyanin content (TAC) expressed as mg cyanidin 3-*O*-glucoside/g dry weight. **^µ^** Extraction yields expressed in percent W/W extraction on the basis of freeze dried material. ^ϕ^ Used as standard antioxidants. Values in the same column marked with the same letter are not significantly different (at *p* < 0.05).

### 2.4. Quantification of Individual Anthocyanins

The major anthocyanins were quantified in the six edible berries, for some of the species for the first time. The order for the sum of the major anthocyanins was: calafate > blueberries > arrayan > meli > murtilla > chequen (Table 3) which is coincident with the trend found for the total anthocyanin content (TAC) (Table 2) measured by a colorimetric method. The HPLC quantification method showed good performance, baseline was good (Figure 2), and the correlation coefficients for the standard curves of the glycosilated standard anthocyanins varied from 0.998 to 0.999. The limits of detection for three representative compounds were 0.08 to 0.12 μg/mL and the limits of quantification were 0.24 to 0.35 μg/mL (Table 4). Repeatability for retention time and peak area was good, relative standard deviations were below 2.00% [34]. As seen in Table 4 all recovery results varied from 97.93 ± 0.33 to 99.72 ± 1.34 and were within the usually required recovery range of 100% ± 5% [34]. However, the anthocyanin concentration in our Chilean blueberries sample is quite different from those published for blueberries from other locations [31,35] being the major anthocyanins found peonidin-3-*O*-arabinoside and delphinidin-3-*O*-arabinoside (37.43 ± 4.76 and 34.43 ± 3.28 mg/100 g fresh weight, respectively) followed by malvidin-3-*O*-glucoside and petunidin-3-*O*-rutinoside (Table 3). In the case of calafate (*Berberis* *microphylla*) the major anthocyanins were delphinidin 3-*O*-galactoside, petunidin-3-*O*-glucoside and malvidin-3-*O*-glucoside (60.42 ± 1.28, 51.39 ± 1.65 and 42.94 ± 1.25, mg/100 g fresh weight, respectively). We found as the major anthocyanin in this species delphinidin 3-*O*-galactoside, but Ruiz *et al* [15] reported delphinidin 3-*O*-glucoside as the major constituent (8.83 ± 1.53 µmol/g fresh weight), followed by petunidin-3-glucoside (4.71 ± 1.08 µmol/g fresh weight). For chequén (*Luma chequen*) the main anthocyanins were cyanidin-3-*O*-galactoside, petunidin-3-*O*-glucoside and petunidin-3-*O*-galactoside (43.46 ± 1.39, 12.83 ± 1.65 and 9.55 ± 1.02 mg/100 g fresh weight, respectively), and for arrayán (*Luma apiculata*) were petunidin-3-*O*-glucoside, malvidin-3-*O*-glucoside, delphinidin 3-*O*-galactoside and cyanidin-3-*O*-glucoside (48.21 ± 2.2, 44.75 ± 3.31, 34.43 ± 2.12 and 9.45 ± 0.15 mg/100 g fresh weight, respectively). Our sample of murtilla (*Ugni molinae*) showed two main anthocyanins (petunidin-3-*O*-rutinoside and peonidin-3-*O*-glucoside, Figure 2, Table 1 and Table 3) and meli (*Amomyrtus meli*) showed six main glycosilated anthocyanins including cyanidin-3-*O*-galactoside and petunidin-3-*O*-galactoside as major ones (Table 1 and Table 3). These compounds were quantified in these *Luma* species for the first time.

### 2.5. Antioxidant Features

The order of the antioxidant activity measured by the bleaching of the radical DPPH and the ferric reducing antioxidant power (FRAP) showed by the six fruits was calafate > blueberry > arrayán > meli > murta > chequén which is also the order found for the sum of the individual major anthocyanins measured by HPLC. A similar trend was observed for superoxide anion scavenging activity (Table 2, Figure S1, Supplementary Material). Calafate showed the highest antioxidant activity (2.33 ± 0.21 µg/mL and 124.46 ± 6.54 µM TE/g dry weight in the DPPH and FRAP assays, respectively, Table 2), followed by blueberry (3.32 ± 0.18 µg/mL and 96.15 ± 5.39 µM TE/g DW), and arrayán (5.88 ± 0.21 and93.4 ± 4.68 µM TE/g DW, Table 2). The bleaching of the radical DPPH for calafate was close to that shown by the standards gallic acid and cyanidin-3-glucoside (1.36 ± 0.22 and 8.47 ± 1.23 µg/mL, respectively). The antioxidant activities showed positive correlation with polyphenolic content assays (0.67 ≥ R^2^ ≥ 0.9856). It is reported that fruits antioxidant activities and composition of phenolics are dependent of genetic differences among different species and environmental conditions and harvest and/or ripeness within the same species [11,36] which can explain the differences in phenolic composition and antioxidant capacities found between the species under study and among other reports of antioxidant activities and phenolic composition of the same species from other zones of Chile [11,12,15].

**Table 3:** Major anthocyanins quantified by HPLC-DAD in six edible berry fruits from the VIII Region of Chile.

	Anthocyanin (mg/100 g) ^a^													
Berry Species	2	3	6	7	8	10	11	13	15	16	17	18	20	Total
*Vaccinium corymbosum*	19.23 ± 3.18	8.51 ± 3.29 a	21.17 ± 0.32	0.96 ± 0.12	14.29 ± 2.15	9.27 ± 2.22 d	1.78 ± 0.01	14.28 ± 0.98	34.43 ± 3.28	nd	16.42 ± 1.45	nd	37.43 ± 4. 76	177.77
*Berberis microphylla*	nd	60.42 ± 1.28	nd	21.89 ± 2.74 b	nd	51.39 ± 1.65 e	6.45 ± 0.89	nd	9.28 ± 0.01	3.96 ± 0.02	42.94 ± 1.25 f	3.84 ± 0.02	nd	200.17
*Luma chequén*	nd	3.72 ± 0.02	43.46 ± 1.39	5.29 ± 0.23	nd	12.83 ± 1.65	9.55 ± 1.02	nd	nd	nd	nd	nd	nd	74.85
*Luma apiculata*	2.6 ± 0.01	34.43 ± 2.12	nd	9.45 ± 0.15	nd	48.21 ± 2.2 e	nd	nd	nd	nd	44.75 ± 3.31 f	nd	nd	139.44
*Ugni molinae*	nd	nd	nd	nd	51.37 ± 0.28	nd	4.87 ± 0.02	nd	nd	61.48 ± 2.42	nd	4.43 ± 0.04	nd	122.15
Amomyrtus meli	nd	8.87 ± 1.76a	48.39 ± 2.23	20.43 ± 2.39 b		13.54 ± 2.46 d	27.12 ± 1.25	nd	nd	nd	8.45 ±1.13	nd	nd	126.80

^a^ Expressed as mg/100 g fresh weight, measurements are expressed as mean ± SD of five parallel determinations. (Values in the same row marked with the same letter are not significantly different at *p* < 0.05). nd: not detected/determined.

**Table 4:** Inter-day and Intra-day accuracy and precision (as RSD%), limits of detection (LOD) and quantification (LOQ) and recovery of three major anthocyanins (compounds **3**, **7** and **10**).

		Inter day			Intra day				
Compound	Nominal concentration (μg/mL)	Observed concentration (μg/mL)	Accuracy (%)	RSD%	Observed concentration (μg/mL)	Accuracy (%)	RSD%	LOD-LOQ (μg/mL)	Sample-Recovery (mean ± RSD% )
**3**	10	10.94 ± 0.05	109.45	0.071	11.83 ± 0.05	118.33	0.04	0.08–0.24	Calafate 97.93 ± 0.33
**3**	20	20.91 ± 0.62	104.55	0.80	21.73 ± 0.37	108.66	0.34		Arrayán 98.73 ± 1.50
**3**	40	40.49 ± 0.70	101.23	0.69	41.2 ± 0.72	103.00	0.70		Blueberry 98.57 ± 0.33
**7**	10	12.0 ± 0.08	120.00	0.08	12.13 ± 0.15	121.33	0.12	0.12–0.35	Calafate 99.72 ± 1.34
**7**	20	19.81± 0.27	99.08	0.28	20.36 ± 0.76	101.80	0.75		Arrayán 98.97 ± 1.98
**7**	40	40.77 ± 0.37	101.93	0.36	40.46 ± 0.67	101.15	0.66		Chequén 99.84 ± 0.16
**10**	10	10.56 ± 0.81	105.66	0.77	9.99 ± 0.12	99.96	0.12	0.09–0.30	Calafate 99.31 ± 0.35
**10**	20	20.20 ± 0.59	101.03	0.58	20.61 ± 0.33	103.05	0.32		Arrayán 98.59 ± 0.38
**10**	40	41.04 ± 0.41	102.64	0.40	40.8 ± 0.36	40.8	0.79		Chequén 98.19 ± 0.76

## 3. Experimental

### 3.1. Chemicals and Plant Material

Folin–Ciocalteu phenol reagent (2 N), reagent grade Na_2_CO_3_, AlCl_3_, HCl, FeCl_3,_ NaNO_2,_ NaOH, quercetin, trichloroacetic acid, sodium acetate, HPLC-grade water, HPLC-grade acetonitrile, reagent grade MeOH and formic acid were obtained from Merck (Darmstadt, Germany) Cyanidin, delphinidin 3-*O*-galactoside, cyanidin-3-*O*-galactoside, cyanidin-3-*O*-glucoside, petunidin-3-*O*-glucoside, petunidin-3-*O*-galactoside, peonidin-3-*O*-galactoside, peonidin-3-*O*-glucoside and malvidin-3-*O*-glucoside (all standards with purity higher than 95% by HPLC) were purchased either from ChromaDex (Santa Ana, CA, USA), Extrasynthèse (Genay, France) or Wuxi Apptec Co. Ltd. (Shangai, China). Gallic acid, TPTZ (2,4,6- tri(2-pyridyl)-s-triazine), Trolox, *tert*-butylhydroperoxide, nitro blue tetrazolium, xanthine oxidase and DPPH (1,1-diphenyl-2-picrylhydrazyl radical) were purchased from Sigma-Aldrich Chemical Co. (St. Louis, MO, USA). All ripe fruits for this study (aprox. 500 g each) were collected at Región del Bio-Bio, Chile. Sampling was performed using sterile disposable gloves and rigid plastic sample containers and each sample was submitted individually by overnight courier to our laboratory in Antofagasta to prevent deterioration. This sampling methodology was previously used for other edible fruits [19,23,33]. Random healthy ripe fruits, representative of the lot, were collected from various specimens (at least 10 fruits per specimen) and different locations (at least 3) in each growing area. Ripe fruits of arrayán *(L. apiculata* (DC.) burret, chequén (*L. chequén* (Molina) A. Gray), and murta (*U. molinae* Turcz) were collected in Re-Re, Chile in May 2011. Meli (A. meli (Phil.) D. Legrand & Kausel and calafate (*B. microphylla* G. Forst) were collected in the Andean woods of Santa Bárbara, in May 2011. Blueberries (*V. corymbosum*) variety highbush Brigitta were collected in April 2011 in the area of Chillán. Voucher herbarium specimens including samples of fruits were deposited at the Laboratorio de Productos Naturales, Universidad de Antofagasta, Antofagasta, Chile, with the numbers La-111505-1, Lc-111505-2, Um-111505-1, Am-111805-1, Bm-111805-1 and Vc-110704-1, respectively.

### 3.2. Sample Preparation

Fresh fruits (Figure S4–S9, supplementary material) were carefully washed, separately homogenized in a blender and freeze-dried (Labconco Freezone 4.5 L, Kansas, MO, USA). Ten grams of each lyophilized fruit was finally pulverized in a mortar, defatted thrice with 100 mL of n-hexane and then extracted with 100 mL of 0.1% HCl in MeOH in the dark in an ultrasonic bath for one hour each time, The extracts were combined, filtered and evaporated *in vacuo* in the dark (40 °C). The extracts were suspended in 20 mL ultrapure water and loaded onto an XAD-7 (100 g) column. The column was rinsed with water (100 mL) and phenolic compounds were eluted with 100 mL of MeOH acidified with 0.1% HCl. This methodology was previously used for other edible fruits [19,23,33]. The solutions were combined and evaporated to dryness under reduced pressure (40 °C) to give 634.20, 739.20, 499.93, 672.24, 489.93 and 521.38 mg of *L. apiculata*, *L. chequén*, *B. microphylla*, *V. corymbosum*, A. meli and *U. molinae* fruits, respectively.

### 3.3. Liquid Chromatography Analysis

A portion of each extract (approximately 2 mg) obtained as explained above was dissolved in 2 mL 0.1% HCl in MeOH, filtered through a 0.45 µm micropore membrane (PTFE, Waters, Milford, MA, USA) before use and was injected into the HPLC-PDA and ESI-ToF-MS equipment. Qualitative HPLC-PDA analysis of the extracts was performed using a Waters Alliance 2695 system equipped with 2695 separation module unit and 2996 PDA detector and a 250 × 4.6 mm, 5 µm, 100 Å, Luna C-18 column (Phenomenex, Torrance, CA, USA), with a linear gradient solvent system of 0.1% aqueous formic acid (solvent A) and acetonitrile 0.1*%* formic acid (solvent B) as follows: 90% solvent A until 4 min, followed by 90%–75% solvent A over 25 min, then 75%–10% A over 35 min, then going back to 90% solvent A until 45 min. and finally reconditioning the column with 90% solvent A isocratic for 15 min. The flow rate and the injection volume were 0.5 mL/min and 20 µL, respectively. The compounds were monitored using a wavelength range of 210–800 nm.

### 3.4. Validation of the HPLC Method

Quantification was done by external standardization, using the respective standard anthocyanins, at the wavelengths of maximum absorption of the compounds. For the validation of the analytical method based on HPLC factors, linearity, precision, detection limits and accuracy were evaluated following [34]. Stock solutions of all seven standard compounds (**3**, **4**, **6**, **7**, **10**, **11**, and **17**) were prepared by dissolving one milligram of each anthocyanin in methanol-formic acid 1% (1 mg/mL). Several calibration levels were prepared by diluting the stock solutions with methanol-formic acid 1% yielding concentrations of 15.65, 31.25, 62.5, 125, 250 and 500 µg/mL. The calibration curves (R^2^ > 0.098) were obtained by plotting peak areas versus concentrations. Compound **15** was quantified using the calibration curve obtained for **3**, compounds **15**–**18** and **20 ** with the calibration curve of **11** and compound **2** with the calibration curve of compound **7**. Limits of detection (LOD) and quantitation (LOQ) were measured for three representative compouns (**3**, **7** and **10**, Table 4) and are reported as the concentrations that gave signal-to-noise ratios of 3 and 10, respectively, from three replicate injections. Accuracy was determined by spiking three standard anthocyanins (**3**, **7** and **10**, Table 4) at three concentration levels (10: low, 20: medium, and 40 µg/mL: high spike) in one gram of each fresh fruits, which was then extracted and assayed as described before. Mean percentage recovery in relation to the theoretically present amounts (% recovery = amount detected × 100/theoretical amount) were used as a measure of accuracy (Table 4). The relative standard deviation (RSD%) within the measurements was considered as a measure of precision and repeatability. The samples were prepared and analyzed for anthocyanin concentration on the same day and on three consecutive days (n = 5) for intra- and interday precision respectively. 

### 3.5. Mass Spectrometric Conditions

Hyphenated PDA with high-resolution electrospray ionization-time of flight-mass spectrometry (HR-ESI-ToF-MS) analysis was performed using a LCT premier XE ToF mass spectrometer (Waters) equipped with an ESI interface and controlled by MassLynx V4.1 software, using the chromatographic conditions as stated above. The compounds were monitored using PDA with a wavelength range of 210–800 nm, while mass spectra were acquired with electrospray ionization and the ToF mass analyzer in both positive and negative modes over the range *m/z*: 100–1000. The capillary voltages were set at 3000 V (positive mode) and 2800 V (negative mode), respectively, and the cone voltage was 20 V. Nitrogen was used as the nebulizer and desolvation gas. The desolvation and cone gas flow rates were 300 and 20 L/h, respectively. The desolvation temperature was 400 °C, and the source temperature was 120 °C. For the dynamic range enhancement (DRE) lockmass, a solution of leucine enkephalin (Sigma–Aldrich, Steinheim, Germany) was infused by a secondary reference probe at 200 pg/mL in CH_3_CN/water (1:1) containing 0.1% formic acid with the help of a second LC pump (Waters 515 HPLC pump). The reference mass was scanned once every five scans for each positive and negative data collection. Both positive and negative ESI data were collected using a scan time of 0.2 s, with an interscan time of 0.01 s, and a polarity switch time of 0.3 s. The full chromatograms were recorded at two different aperture voltages. The most intense fragmental ions and molecular ions could be obtained, when the aperture voltage were set at 60 V and 0 V, respectively. V-optics mode was used for increased intensity. 

### 3.6. Antioxidant Assays

#### 3.6.1. Free Radical Scavenging Capacity

The free radical scavenging capacity of the extracts was determined by the DPPH^.^ assay as previously described [37], with some modifications. DPPH radical absorbs at 517 nm, but upon reduction by an antioxidant compound its absorption decreases. Briefly, 50 µL of processed SPE MeOH extract or pure compound prepared at different concentrations was added to 2 mL of fresh 0.1 mM solution of DPPH in methanol and allowed to react at 37 °C in the dark. After thirty minutes the absorbance was measured at 517 nm. The DPPH scavenging ability as percentage was calculated as: DPPH scavenging ability = (A_control_ − A_sample_/A_control_) × 100. Afterwards, a curve of % DPPH bleaching activity versus concentration was plotted and IC_50_ values were calculated. IC_50_ denotes the concentration of sample required to scavenge 50% of DPPH free radicals. The lower the IC_50_ value the more powerful the antioxidant activity. Gallic acid (from 1.0 to 125.0 µg/mL, R^2^ = 0.991) and cyanidin 3-*O*-glucoside (from 1.0 to 125.0 µg/mL, R^2^ = 0.997) were used as standard antioxidant compounds.

#### 3.6.2. Ferric Reducing Antioxidant Power

The determination of ferric reducing antioxidant power or ferric reducing ability (FRAP assay) of the extracts was performed as described by [38] with some modifications. The stock solutions prepared were 300 mM acetate buffer pH 3.6, 10 mM TPTZ (2,4,6-tri(2-pyridyl)-s-triazine) solution in 40 mM HCl, and 20 mM FeCl_3_·6H_2_O solution. Plant extracts or standard methanolic Trolox solutions (150 µL) were incubated at 37 °C with 2 mL of the FRAP solution (prepared by mixing 25 mL acetate buffer, 5 mL TPTZ solution, and 10 mL FeCl_3_·6H_2_O solution) for 30 min in the dark. Absorbance of the blue ferrous tripyridyltriazine complex formed was then read at 593 nm. Quantification was performed using a standard calibration curve of the antioxidant Trolox (from 0.2 to 2.5 µmol/mL, R^2^: 0.995). Samples were analyzed in triplicate and results are expressed in µmol TE/gram dry mass. 

#### 3.6.3. Superoxide Anion Scavenging Activity

The enzyme xanthine oxidase is able to generate superoxide anion radical (O_2_**^.−^**) “*in vivo*” by oxidation of reduced products from intracellular ATP metabolism. The superoxide anion generated in this reaction sequence reduces the nitro blue tetrazolium dye (NBT), leading to a chromophore with a maximum of absorption at 560 nm. Superoxide anion scavengers reduce the speed of generation of the chromophore. The superoxide anion scavenging activities of isolated compounds and fractions were measured spectrophotometrically in a microplate reader as reported previously [23]. All compounds, and berry extracts were evaluated at 100 μg/mL. Values are presented as mean ± standard deviation of three determinations. 

#### 3.6.4. Polyphenol, Flavonoids and Anthocyanin Contents

The total polyphenolic contents (TPC) of *Luma* fruits and leaves were determined by the Folin-Ciocalteau method [19,33,39] with some modifications. An aliquot of each processed SPE extract (200 μL, approx. 2 mg/mL) was added to the Folin–Ciocalteau reagent (2 mL, 1:10 v/v in purified water) and after 5 min of reaction at room temperature (25 °C), 2 mL of a 100 g/l solution of Na_2_CO_3_ was added. Sixty minutes later the absorbance was measured at 710 nm. The calibration curve was performed with gallic acid (concentrations ranging from 16 to 500 μg/mL, R^2^ = 0.999) and the results were expressed as mg gallic acid equivalents/g dry mass. Determination of total flavonoid content (TFC) of the methanolic extracts was performed as reported previously [40] using the AlCl_3_ colorimetric method. Quantification was expressed by reporting the absorbance in the calibration graph of quercetin, which was used as a standard (from 0.1 to 65.0 μg/mL, R^2^ = 0.994). Results are expressed as mg quercetin equivalents/g dry weight. The assessment of total anthocyanin content (TAC) was carried out by the pH differential method according to AOAC as described by [38,41]. Absorbance was measured at 510 and 700 nm in buffers at pH 1.0 and 4.5. Pigment concentration is expressed as mg cyanidin 3-glucoside equivalents/g dry mass and calculated using the formula:

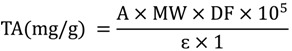

where A = (A510 nm − A700 nm) pH 1.0 − (A510 nm − A700 nm) pH 4.5; MW (molecular weight) = 449.2 g/mol; DF = dilution factor; 1 = cuvette pathlength in cm; ε = 26,900 L/mol.cm, molar extinction coefficient for cyanidin 3-*O*-β-d-glucoside. 10^3^: factor to convert g to mg. All spectrometric measurements were performed using a Unico 2800 UV-Vis spectrophotometer (Unico Instruments Co. Ltd., Shanghai, China).

### 3.7. Statistical Analysis

The statistical analysis was carried out using the originPro 9.0 software packages (Originlab Corporation, Northampton, MA, USA). The determination was repeated at least three times for each sample solution. Analysis of variance was performed using ANOVA. Significant differences between means were determined by Tukey comparison test (*p* values < 0.05 were regarded as significant). 

## 4. Conclusions

Thirty one anthocyanins, three phenolic acids (feruloylquinic acid, chlorogenic and neochlorogenic acid) and six flavonols (rutin, quercetin, myricetin, hyperoside, isoquercitrin and isorhamnetin) were identified for the first time in six edible berries from the VIII region of Chile using ToF-MS. Among the 31 anthocyanins identified in the six berries under study, twenty three compounds were detected in blueberry, fourteen in calafate, nine in arrayán and six were present in meli, chequén and murta. The anthocyanins detected were mainly branched 3-*O*-glycoconjugates of malvidin, delphinidin, peonidin, petunidin and cyanidin. However, significant differences in the amount of anthocyanins, (which were measured individually by HPLC for the major ones and by TAC colorimetric method) were found for the six berries, which presented also different antioxidant capacities. Blueberry fruits showed the most complex anthocyanin profile, while the fruits of chequen and murta showed a simpler pattern with only six anthocyanins, whereas arrayán and chequén showed a more complex pattern. However, the fruits of calafate (*B. microphylla*) presented the highest antioxidant features and polyphenolic content followed by the fruits of Chilean blueberries (*V. corymbosum*), arrayán (*L. apiculata*) and meli (*A. meli*), which makes calafate, arrayán and meli the better candidates for industrial crop production and potential use in functional foods and nutraceuticals.

## Supplementary Materials

Supplementary materials can be accessed at: http://www.mdpi.com/1420-3049/19/8/10936/s1.

## Supplementary Files

Supplementary File 1
