# Supplementary material for: Anthocyanin Characterization, Total Phenolic Quantification and Antioxidant Features of Some Chilean Edible Berry Extracts
Source: Molecules. 2014 Jul 28;19(8):10936–55. doi: 10.3390/molecules190810936 (PMC6271127; doi:10.3390/molecules190810936)

Supplementary Materials

Figure S1. Antioxidant capacities of six Chilean berries from the VIII region of Chile. (**a**) DPPH scavenging assay; (**b**) Ferric reducing antioxidant power assay; and (**c**) Superoxide anion scavenging assay.

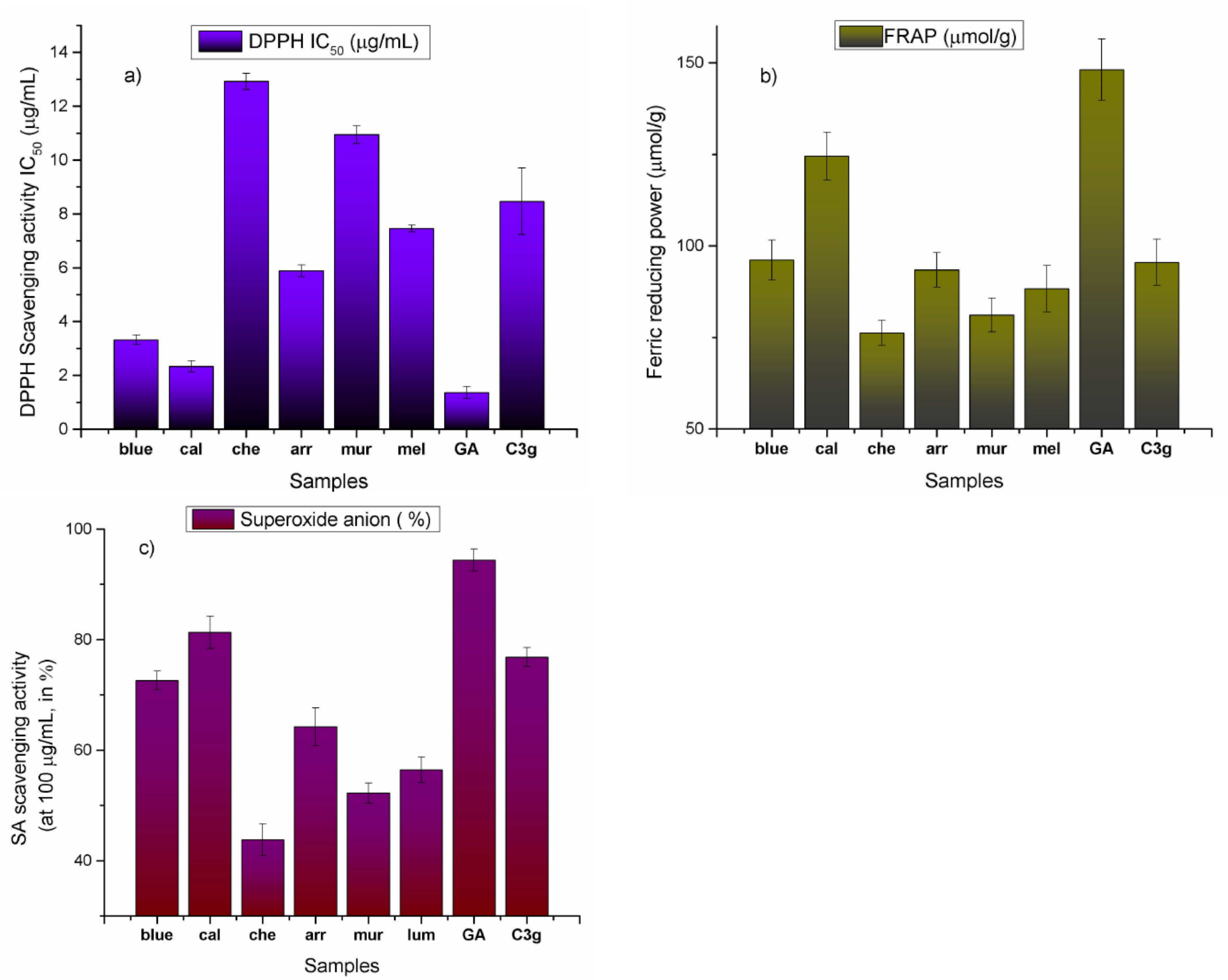

Figure S2. Full scan ToF MS spectra of peaks **3**, **8**, **9** and **10**.

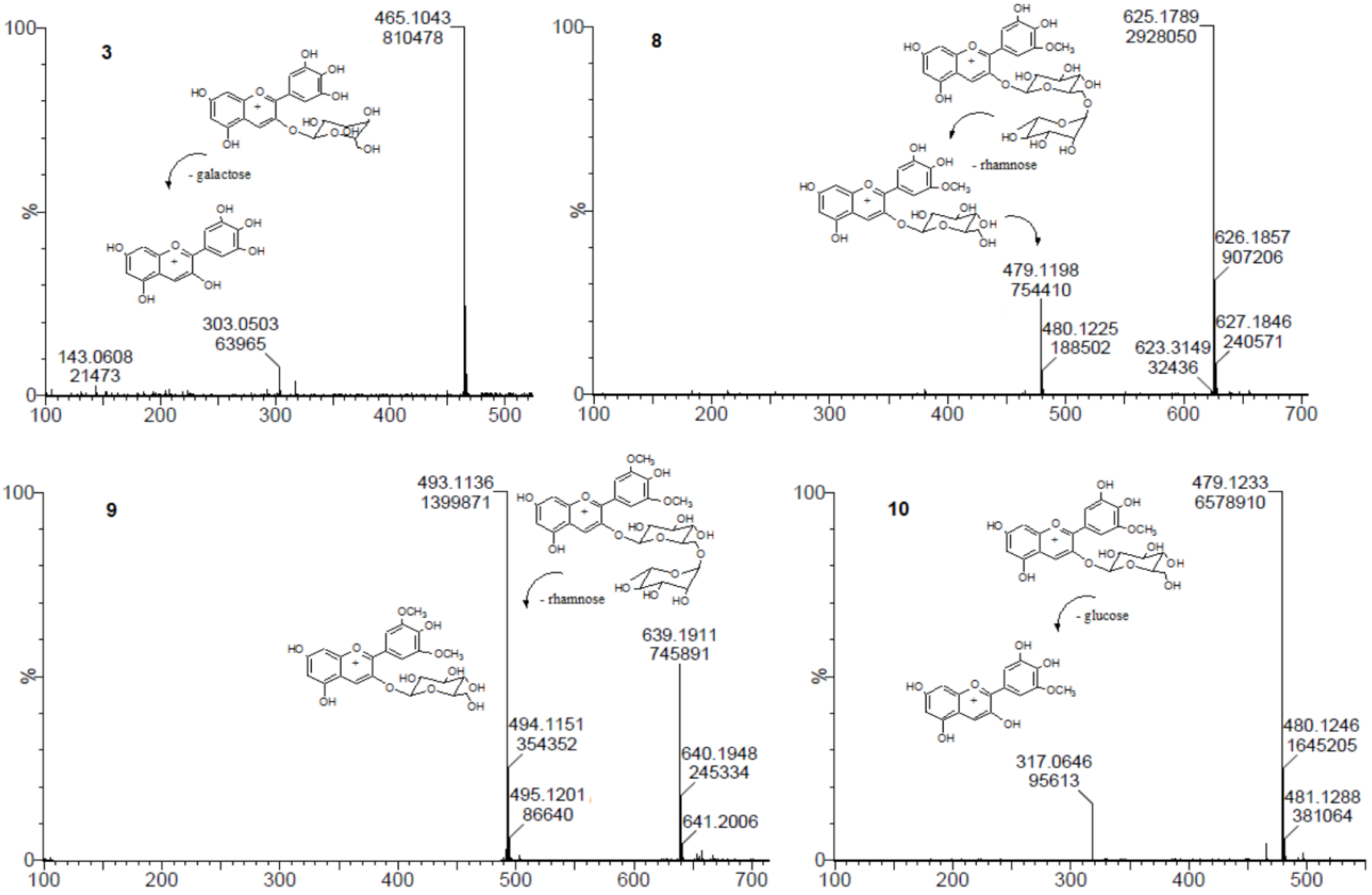

Figure S3. Full scan ToF MS spectra of peaks **16**, **17**, **21**, **22** and **28**.

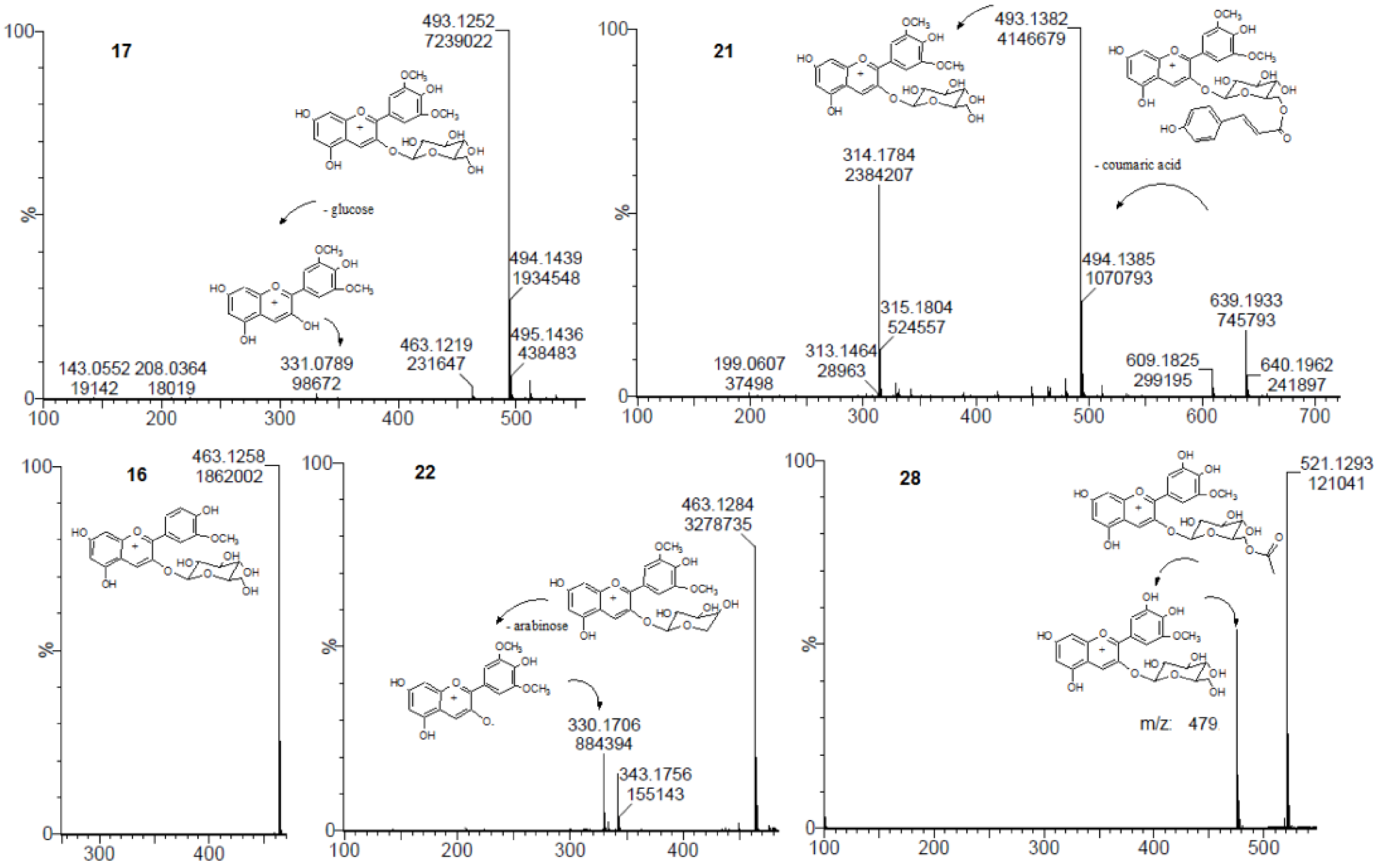

Figure S4. Arrayán sample.

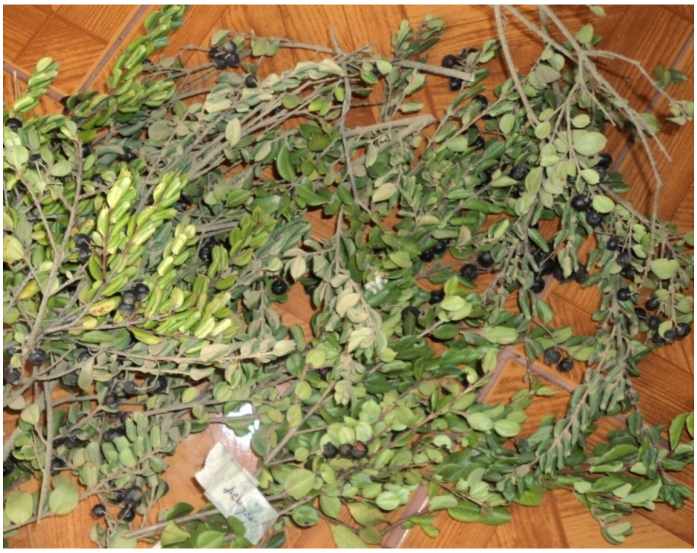

Figure S5. Chequén sample.

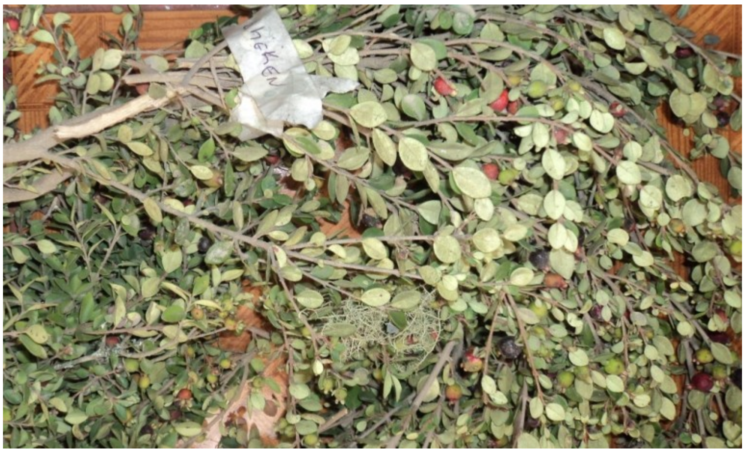

Figure S6. Murtilla sample.

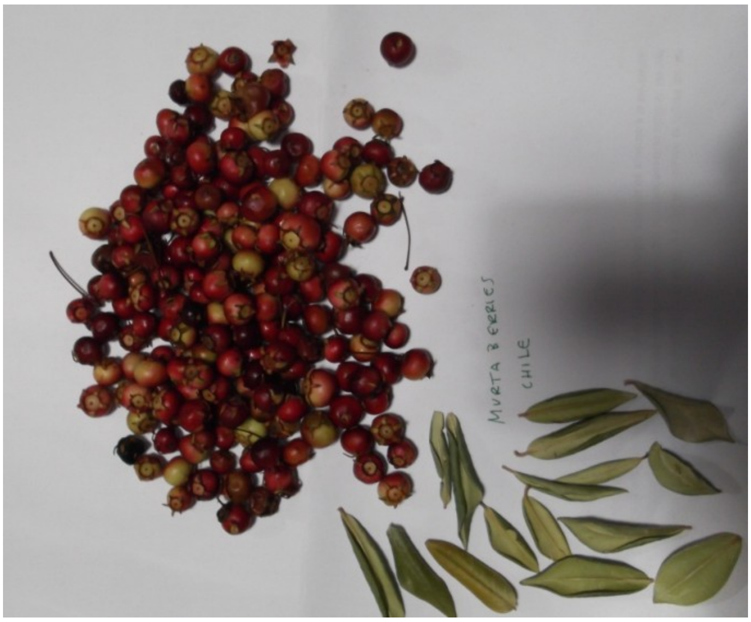

Figure S7. Calafate sample.

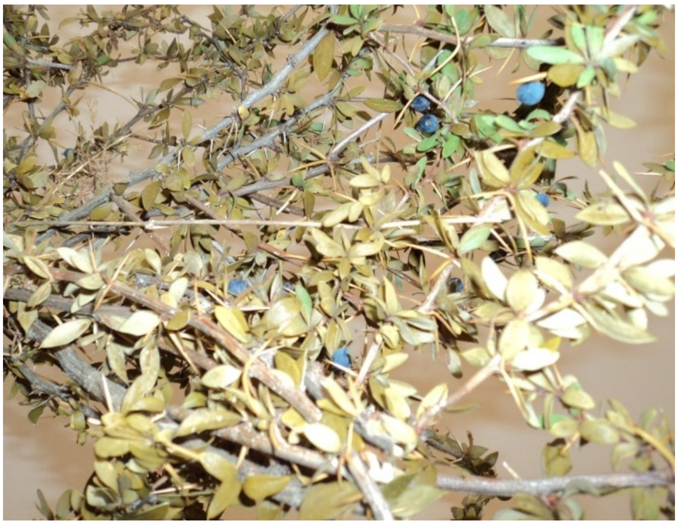

Figure S8. Meli sample.

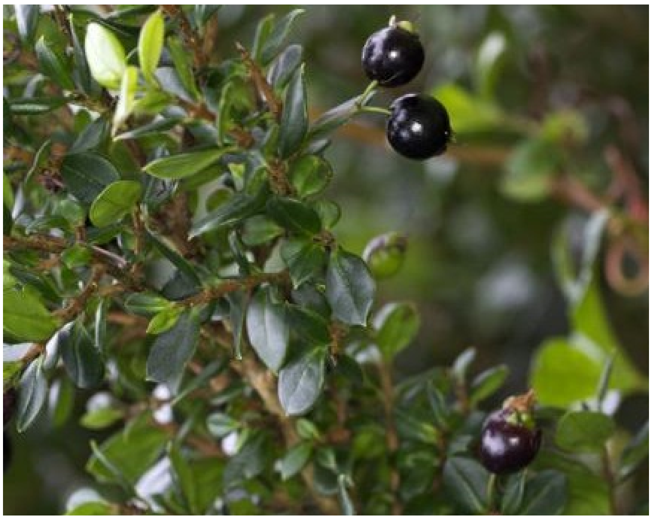

Figure S9. Blueberry sample.

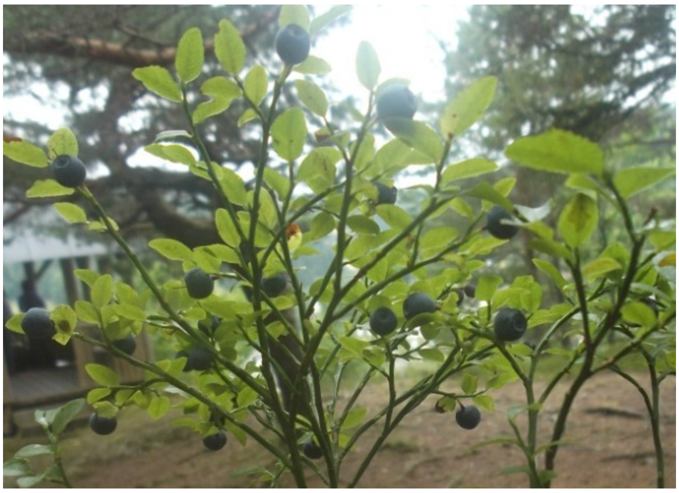

Supplement: Supplementary File 1 [file molecules-19-10936-s001.pdf]
